# From Ultrasound to Histopathology: Agreement Analysis and Depth Correction Model for Basal Cell Carcinoma Using a Portable 20 MHz High-Frequency Ultrasound Device

**DOI:** 10.3390/diagnostics16070978

**Published:** 2026-03-25

**Authors:** Cristina Popescu, Carmen Andrada Iliescu, Andreea Mihaela Truica, Liliana Gabriela Popa, Andrei Ludovic Porosnicu, Irina Tudose, Carmen Boeru, Marius Nicolae Popescu

**Affiliations:** 1 Department of Oncologic Dermatology, Elias Emergency University Hospital, Carol Davila University of Medicine and Pharmacy, 020021 Bucharest, Romania; cristina.popescu25@umfcd.ro (C.P.); liliana.popa@umfcd.ro (L.G.P.); 2 Clinic of Dermatology, Elias Emergency University Hospital, 011461 Bucharest, Romania; 3 Department of Plastic and Reconstructive Surgery, Elias Emergency University Hospital, Carol Davila University of Medicine and Pharmacy, 020021 Bucharest, Romania; andrei.porosnicu@umfcd.ro; 4 Histopathology Department, Elias University Emergency Hospital, 011461 Bucharest, Romania; irina_tds@yahoo.com; 5 Faculty of Medicine, Carol Davila University of Medicine and Pharmacy, 020021 Bucharest, Romania; carmen-ioana.boeru0720@stud.umfcd.ro; 6 Department of Physical and Rehabilitation Medicine, Elias Emergency University Hospital, Carol Davila University of Medicine and Pharmacy, 020021 Bucharest, Romania; marius.popescu@umfcd.ro

**Keywords:** basal cell carcinoma, high-frequency ultrasound, tumor depth, Bland–Altman analysis, non-invasive imaging

## Abstract

**Background:** High-frequency ultrasound (HFUS) represents a valuable non-invasive imaging modality for assessing cutaneous tumors. In basal cell carcinoma (BCC), preoperative estimation of tumor depth may support therapeutic decision-making and surgical planning. However, agreement between HFUS-derived and histopathologic depth measurements remains incompletely characterized, particularly with 20 MHz probes in routine clinical practice. **Objectives:** To evaluate agreement between 20 MHz HFUS and histopathology for BCC tumor depth using Bland–Altman analysis and to derive a preliminary correction equation to estimate histologic depth from HFUS measurements. **Methods:** This prospective observational pilot study included 15 patients with 16 histologically confirmed BCC lesions. All lesions underwent preoperative 20 MHz HFUS followed by surgical excision, with HFUS-derived tumor depth compared with histopathologic depth. Agreement was assessed using Bland–Altman analysis, and linear regression was performed to derive a preliminary correction equation. **Results:** Sixteen BCC lesions were analyzed. The mean difference between HFUS and histopathologic tumor depth was −0.07 mm, with 95% limits of agreement from −1.58 to +1.45 mm. HFUS and histopathologic depth measurements were highly correlated (*R*^2^ = 0.99). A correction equation was derived: estimated histopathologic depth (mm) = −0.52 + 1.10 × HFUS depth (mm). **Conclusions:** Twenty MHz HFUS demonstrated good agreement with histopathology for tumor depth assessment in BCC, with clinically acceptable variability. The proposed correction equation may improve interpretation of HFUS measurements; however, further validation in larger cohorts is required.

## 1. Introduction

Basal cell carcinoma (BCC) is the most prevalent form of skin cancer worldwide and occurs more often than all the other human malignancies combined [1,2]. Given the growing elderly population and the cumulative effect of lifetime ultraviolet exposure, incidence rates have been rising over recent decades and are projected to keep rising through at least 2040 [3]. Increasing incidence has important public health implications and places a substantial burden on dermatologic healthcare systems [4,5]. BCC shows an impressive potential for progressive local invasion, particularly in anatomically and functionally critical sites [6,7]. This can lead to considerable morbidity, despite the rarity of metastases [8]. Therefore, an accurate assessment and appropriate treatment planning, starting from early stages, are required.

Tumor depth represents an extremely relevant parameter influencing management strategies in BCC [9,10]. Superficial lesions may be amenable to non-surgical therapies, whereas deeper or more aggressive tumors typically require surgical excision, often with margin-controlled techniques [11,12]. Traditionally, tumor depth is determined by histopathologic examination following excision. While histopathology remains the reference standard, it provides retrospective information and may be affected by multiple factors including sampling error, tissue shrinkage during processing, and sectioning plane variability [13,14,15].

High-frequency ultrasound (HFUS) has emerged as a non-invasive imaging technique capable of visualizing cutaneous structures with high axial resolution [16]. HFUS allows real-time evaluation of tumor morphology, depth and extent prior to intervention, providing valuable information, which facilitates a more precise treatment strategy [17,18]. This may help reduce incomplete excision and subsequent recurrence, thereby preserving both functional and cosmetic outcomes, by limiting defect size or the extent of reconstruction [19,20]. Several studies have demonstrated strong correlations between HFUS-measured and histologic tumor depth in BCC [21,22]. However, correlation alone does not establish interchangeability between methods [23]. Agreement analysis is required to determine whether HFUS can reliably approximate histologic measurements within clinically acceptable limits.

Furthermore, systematic differences between HFUS and histopathologic measurements have been reported [20,24], raising the possibility that a correction factor may improve the clinical interpretability of HFUS-derived data. Evidence addressing this aspect remains limited, particularly for 20 MHz probes, which are widely available in routine dermatologic practice.

The present study aimed to evaluate agreement between 20 MHz HFUS and histopathology for tumor depth assessment in BCC using Bland–Altman analysis and to derive a preliminary correction equation to improve clinical interpretability of HFUS measurements. By addressing persistent, modality-related measurement bias and bringing HFUS-derived tumor depth closer to the histopathologic reference, this correction equation may better support preoperative decision-making.

## 2. Materials and Methods

### 2.1. Study Design

A prospective, single-center, observational pilot study was conducted over a six-month period (June–December 2025) at the Oncological Dermatology Department of Elias University Emergency Hospital, Bucharest, Romania, a tertiary referral center for the diagnosis and surgical treatment of cutaneous malignancies, in collaboration with the Plastic Surgery Department of the same institution. Adult patients (≥18 years) presenting with cutaneous lesions demonstrating clinical and dermoscopic features consistent with BCC were evaluated for study eligibility. Lesions meeting established clinical criteria for surgical management underwent standard excision, in accordance with routine dermatologic practice. Histopathologic examination of the excised specimens provided definitive diagnostic confirmation and only histopathologically confirmed BCC lesions were included in the final analysis. Enrollment was restricted to lesions allowing adequate HFUS image acquisition for reliable assessment and histopathological confirmation of BCC following excision.

Exclusion criteria comprised previous treatment of the target lesion (surgical, topical, photodynamic, or radiotherapeutic), recurrent BCC at the same site, and site-related or technical factors precluding ultrasonographic evaluation. Selected lesions were evaluated using a predefined diagnostic workflow comprising clinical assessment, 20 MHz HFUS imaging, and histopathological examination of the excised specimen. One patient presented with two anatomically distinct BCC lesions, which were analyzed as separate cases, resulting in a total of 16 lesions from 15 patients.

Ethical approval for this study was obtained from the Institutional Ethics Committee of the Elias University Emergency Hospital, Bucharest (Approval No. 16052025-1, date of approval: 16 May 2025). The study was conducted in accordance with the Declaration of Helsinki. Written informed consent was obtained from all subjects involved in the study, including consent for publication of images.

### 2.2. High-Frequency Ultrasound Examination

HFUS was performed prior to surgical excision using a portable handheld 20 MHz high-definition linear transducer (Clarius L20 HD3, Clarius Mobile Health, Vancouver, Canada) by one of two dermatologists experienced in cutaneous ultrasonography, according to a standardized acquisition protocol; inter-operator repeat examinations were not performed. Standard acoustic coupling gel was applied between the probe and the skin surface, and care was taken to minimize probe pressure during image acquisition in order to avoid compression of superficial structures. Each lesion was examined in two orthogonal planes (longitudinal and transverse).

Tumor depth was the primary HFUS measurement and was defined as the maximum vertical distance from the epidermal surface to the deepest hypoechoic tumor boundary. Measurements were obtained with the ultrasound beam oriented perpendicular to the skin surface and were recorded in millimeters. For each lesion, depth was measured on the image demonstrating the greatest vertical tumor extent.

In addition to tumor depth, lesions were assessed descriptively for selected ultrasonographic characteristics, including shape, internal echogenicity/echotexture, and vascularity evaluated using both color and power Doppler modes. These features were recorded for descriptive purposes and selection of representative images and were not included in the analysis.

### 2.3. Histopathologic Assessment

Surgical excision was performed using conventional en bloc excision with predefined lateral safety margins of 4–5 mm. Excision was carried to an appropriate deep plane to ensure complete tumor removal. Specimens were fixed in formalin and processed according to standard histopathology protocols, including paraffin embedding, sectioning, and hematoxylin–eosin staining.

All slides were reviewed by a senior dermatopathologist who was fully blinded to ultrasound-derived measurements and imaging data. Histopathological evaluation recorded tumor subtype and routine parameters, including maximum lesion size, level of invasion, tumor depth and the presence of associated inflammatory infiltrate.

Tumor depth was measured on histopathological sections as the maximum perpendicular distance from the epidermal surface to the deepest identifiable tumor nest. In ulcerated lesions, measurements were referenced to the base of the ulcer. Depth was recorded on the section demonstrating the greatest depth of invasion, in accordance with routine dermatopathological practice.

### 2.4. Statistical Analysis

Statistical analyses were performed using Python (version 3.10). Continuous variables are presented as mean ± standard deviation. Agreement between HFUS and histopathologic tumor depth measurements was assessed using Bland–Altman analysis, including calculation of the mean difference (bias) and the 95% limits of agreement.

A simple linear regression model based on ordinary least squares was fitted to estimate histopathologic tumor depth from HFUS measurements, with histopathologic depth as the dependent variable and HFUS-derived depth as the independent variable. Model performance was evaluated using the coefficient of determination (*R*^2^), mean absolute error (MAE), and root mean square error (RMSE). A *p*-value < 0.05 was considered statistically significant.

## 3. Results

### 3.1. Study Population and Lesion Characteristics

The study population consisted of 15 patients with 16 histopathologically confirmed BCC lesions. One patient presented with two anatomically distinct tumors, which were considered separate lesions for analysis. The cohort comprised nine males and six females, with a mean age of 70.31 ± 10.78 years. Lesions were predominantly located in the head and neck region, consistent with the typical anatomical distribution of BCC in older patients.

### 3.2. Tumor Depth Measurements by HFUS and Histopathology

Tumor depth measurements obtained by HFUS and histopathology are summarized in Table 1. The mean HFUS-measured tumor depth was 5.63 ± 5.01 mm, while the mean histopathologic tumor depth was 5.70 ± 5.56 mm. The similarity of these mean values suggests no substantial systematic difference at the cohort level in this pilot sample.

**Figure 1:**
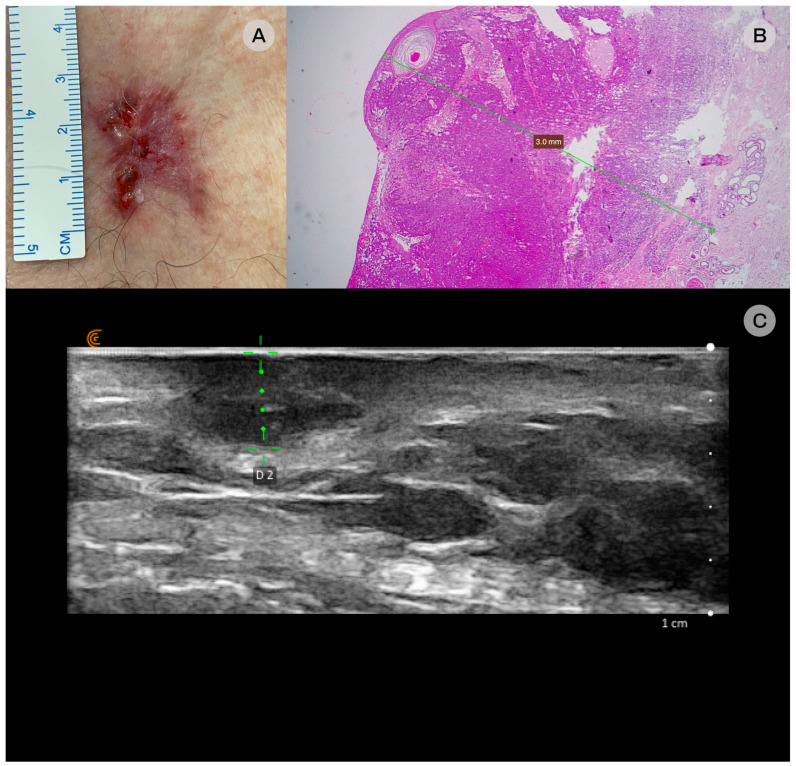
Adenoid basal cell carcinoma (BCC). (**A**) Clinical: an infiltrated plaque with surface ulceration and prominent telangiectasias is present on the anterior thoracic wall. (**B**) Histopathologic examination: the lesion was confirmed to be adenoid BCC and infiltrated the deep dermis, with a maximum depth of 3 mm, hematoxylin-eosin staining, original magnification 25×. (**C**) Ultrasonographic: a well-defined hypoechoic lesion is identified within the subcutaneous tissue, located superficial to the pectoralis major muscle, with no sonographic evidence of muscular or osseous involvement and a maximum depth of 3.5 mm. The underlying hyperechoic structure with posterior acoustic shadowing corresponds to the third parasternal rib.

**Figure 2:**
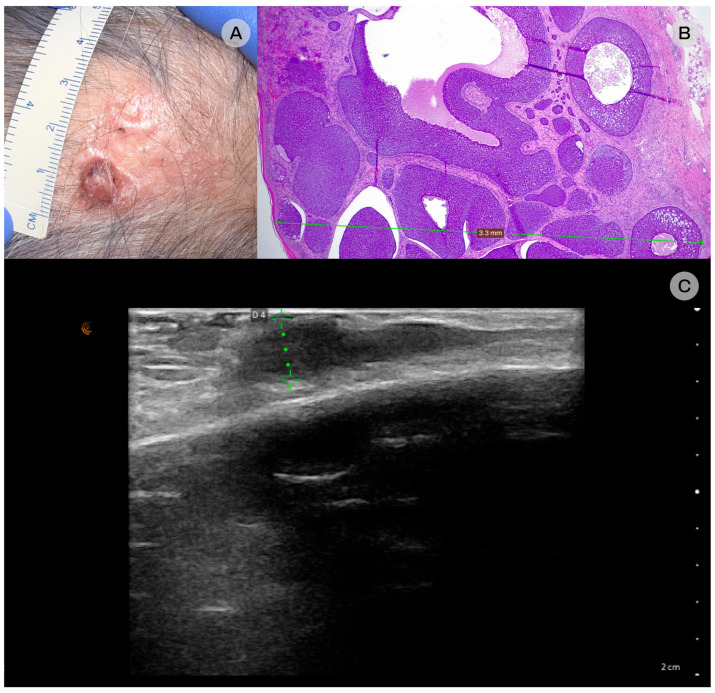
Nodular basal cell carcinoma (BCC). (**A**) Clinical: a pink, shiny, infiltrated plaque located on the scalp featuring an ulcerated nodule and visible telangiectatic vessels. (**B**) Histopathologic examination: the lesion was confirmed to be nodular BCC and infiltrated the reticular dermis, with a maximum depth of 3 mm, hematoxylin-eosin staining, original magnification 25×. (**C**) Ultrasonographic: an oval-shaped, well-demarcated, hypoechoic lesion found within the dermal–subcutaneous (dermo-hypodermic) plane, with lateral, linear extension and a maximum depth of 3.3 mm is identified on the scalp.

**Figure 3:**
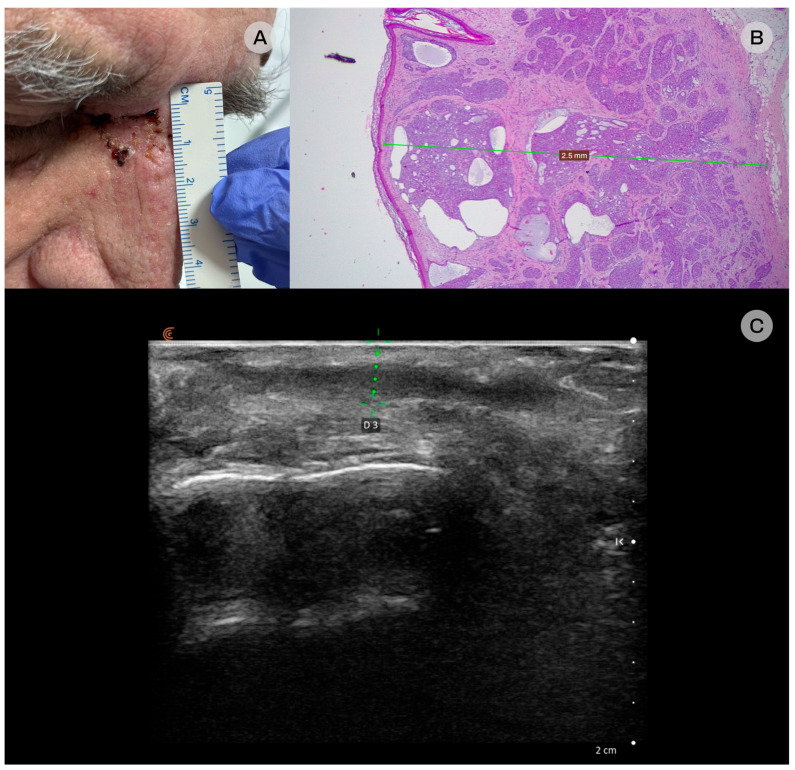
Infiltrative basal cell carcinoma (BCC). (**A**) Clinical: a pink, infiltrated plaque showing multiple ulcerations with hemorrhagic crusts. (**B**) Histopathologic examination: the lesion was confirmed to be infiltrative BCC and infiltrated the deep dermis, with a maximum depth of 2.5 mm on the examined sections, hematoxylin-eosin staining, original magnification 25×. (**C**) Ultrasonographic: a fusiform, well-demarcated hypoechoic lesion, measuring 3.1 in depth, located at the dermal–subcutaneous (dermo-hypodermic) plane is seen at the medial canthus. No sonographic evidence of involvement of the underlying osseous plate.

At the individual lesion level, HFUS both underestimated and overestimated histopathologic depth, with no consistent directional pattern across the range of tumor depth (Figure 1, Figure 2 and Figure 3).

### 3.3. Agreement Analysis Between HFUS and Histopathology

**Figure 4:**
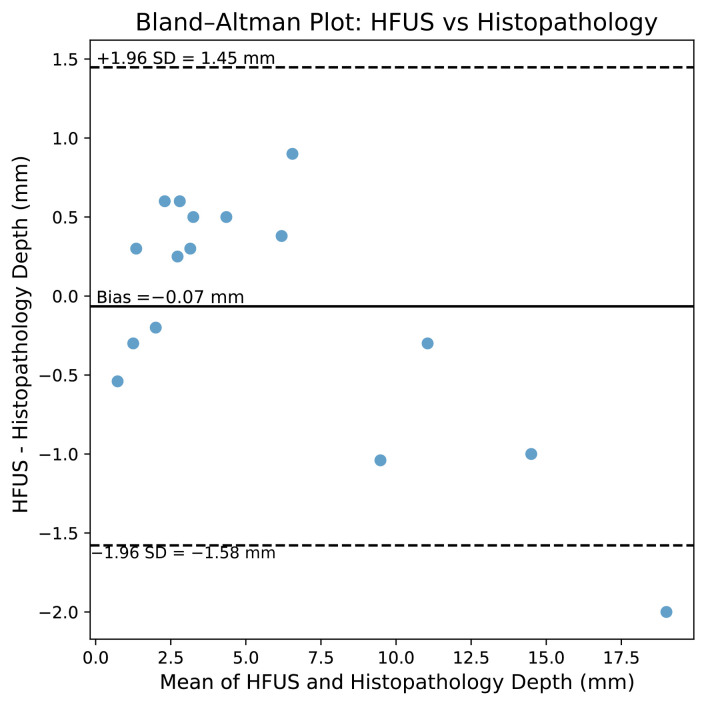
Bland–Altman plot comparing tumor depth measurements obtained by high-frequency ultrasound (HFUS) and histopathologic examination. The difference between measurements (HFUS–histopathology) is plotted against the mean of the two methods for each lesion (*n* = 16 lesions from 15 patients; one patient contributed two lesions). The solid line represents the mean difference (bias) and the dashed lines indicate the 95% limits of agreement (bias = −0.07 mm; limits of agreement: −1.58 to 1.45 mm).

Agreement between HFUS and histopathologic tumor depth measurements was assessed using Bland–Altman analysis (Figure 4). The mean difference (bias) was −0.07 mm, indicating minimal systematic underestimation of tumor depth by HFUS compared with histopathology. The standard deviation of the differences was 0.77 mm, yielding 95% limits of agreement −1.58 mm to +1.45 mm.

These limits indicate that differences between HFUS and histopathologic tumor depth measurements would be expected to fall within approximately ±1.5 mm for most observations. Visual inspection of the Bland–Altman plot revealed no apparent proportional bias, with no clear trend toward increasing disagreement at greater tumor depths, suggesting consistent agreement across the range of measured lesion depths.

### 3.4. Linear Regression Analysis and Correction Equation

**Figure 5:**
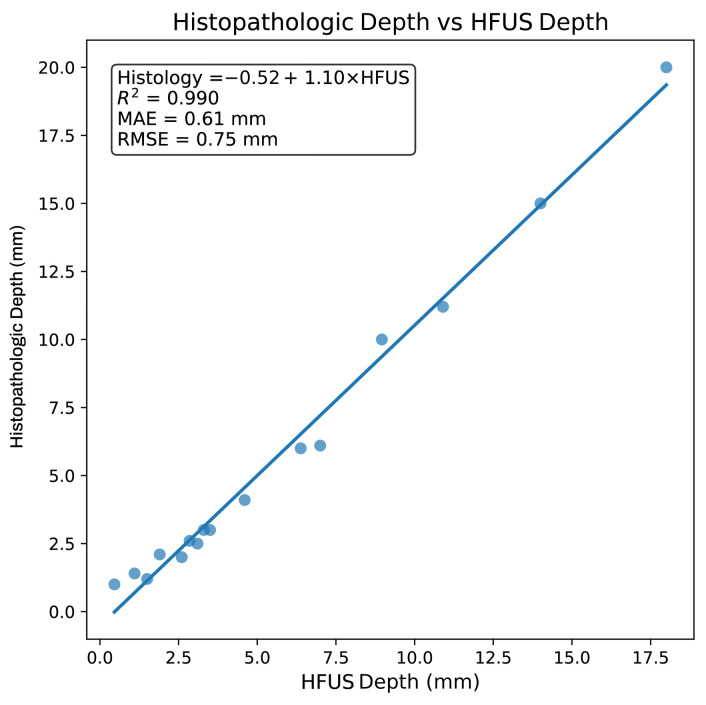
Scatter plot illustrating the relationship between high-frequency ultrasound (HFUS)-measured tumor depth and histopathologic tumor depth across all analyzed lesions. Each point represents an individual lesion and the solid line indicates the fitted linear regression model used to derive the correction equation for estimating histopathologic depth from HFUS measurements.

Linear regression analysis was performed to further assess the relationship between HFUS-measured tumor depth and histopathologic tumor depth (Figure 5). A strong linear association was observed, with a coefficient of determination (*R*^2^) of 0.99, indicating that HFUS depth accounted for approximately 99% of the variability in histopathologic depth.

Based on the linear regression model, a correction equation was derived to estimate histopathologic tumor depth from HFUS measurements:

(1) Estimated histopathologic depth (mm) = −0.52 mm + 1.10 × HFUS depth (mm)

The predictive performance of the model was further evaluated, yielding a mean absolute error (MAE) of 0.61 mm and a root mean square error (RMSE) of 0.75 mm, reflecting good agreement between predicted and observed histopathologic depths within this pilot cohort.

## 4. Discussion

The present prospective pilot study demonstrates that 20 MHz HFUS provides a reliable and clinically meaningful estimation of BCC tumor depth when compared with histopathology. Using an agreement-based analytical approach, we observed minimal systematic bias and narrow limits of agreement between the two methods, supporting the potential role of standard HFUS as a non-invasive tool for preoperative tumor depth assessment in routine oncologic dermatology practice [25].

A key strength of this study is the application of Bland–Altman analysis [26], which provides a clinically interpretable evaluation of agreement between 20 MHz HFUS and histopathology by quantifying systematic bias and the expected range of between-method differences. In contrast to correlation metrics, which primarily reflect whether two measurements vary in parallel, agreement analysis directly addresses the practical question of how closely HFUS-derived depth approximates histologic depth in individual lesions [27]. In our cohort, the mean bias was negligible (−0.07 mm), and the 95% limits of agreement (−1.58 to +1.45 mm) indicate limited dispersion of differences across the observed depth range. Importantly, defining this measurement uncertainty strengthens the clinical interpretability of HFUS and may support informed preoperative counseling and individualized planning, particularly in anatomically sensitive sites and in cases where depth is a determinant of the management strategy.

Our findings are consistent with early foundational work by Bobadilla et al. [28], who reported close correspondence between ultrasound-measured and histologic tumor depth in facial BCCs using HFUS. Beyond this initial evidence, a growing body of literature has explored the role of HFUS in the non-invasive assessment of BCC, including tumor detection, margin delineation and preoperative depth estimation, highlighting the clinical value of ultrasound-derived tumor depth for surgical planning and risk stratification [29,30,31]. However, much of the existing evidence relies predominantly on correlation-based metrics (Pearson correlation coefficients or intraclass correlation coefficients), which evaluate association or consistency, but do not quantify the magnitude of between-method disagreement for individual lesions [14,32,33]. By contrast, the present study extends this literature by applying an agreement-based framework and demonstrating that, even with negligible mean bias, clinically relevant variability persists between HFUS and histopathology, supporting the use of a depth-correction equation to reduce individual-level measurement error and improve preoperative accuracy.

More recent investigations using HFUS at comparable frequencies further support our results. In this context, Barcaui et al. demonstrated high sensitivity and accuracy of 22 MHz HFUS for detecting deep tumor margins and reported a strong correlation between ultrasound-derived and histopathologic depth measurements [34]. Importantly, they identified peritumoral inflammation, fibrosis, adnexal structures and collagen degeneration as major contributors to ultrasound overestimation, findings that are consistent with observations from other HFUS-histopathology correlation studies [35,36,37]. In contrast, underestimation has been reported when the deep margin is poorly delineated (e.g., infiltrative growth beyond the hypoechoic tumor area), when acoustic attenuation or shadowing obscures the interface, or when limited contrast with the surrounding dermis reduces boundary detectability [38,39]. Taken together, these effects provide a biologically plausible basis for the bidirectional discrepancies observed in individual cases and suggest that disagreement is largely biological and tissue-processing-related, rather than reflecting random measurement error or limitations in nominal ultrasound resolution.

The increasing availability of ultra-high-frequency ultrasound (UHFUS) has prompted debate as to whether progressively higher probe frequencies necessarily translate into superior clinical performance for BCC depth assessment [40]. Studies employing 70–75 MHz probes, most notably that of Chauvel-Picard et al., have demonstrated excellent agreement and reproducibility between ultrasound-derived and histopathologic tumor depth measurements, including across a wide range of histologic BCC subtypes [41]. Nevertheless, these investigations consistently report small but persistent differences between ultrasound and histologic measurements [22]. Such discrepancies have been primarily attributed to biological and processing-related factors, including tissue shrinkage during formalin fixation and histologic processing [42], as well as the inclusion of peritumoral stromal reaction and inflammatory changes within the hypoechoic tumor boundary on ultrasound imaging [31]. The persistence of these differences despite the use of UHFUS suggests that perfect concordance between ultrasound and histopathology may be inherently constrained by biological tissue characteristics and histopathologic reference standards, rather than by ultrasound resolution alone [41]. In this context, methodological approaches aimed at quantifying systematic bias and applying depth-correction models may offer greater clinical value than further increases in nominal ultrasound frequency, supporting the rationale for the agreement-based correction strategy proposed in the present study.

The frequency-dependent performance of HFUS has been clearly demonstrated by Khlebnikova et al., who compared 30 MHz and 75 MHz probes and showed that higher frequencies provide superior accuracy for very thin tumors, whereas lower frequencies are more suitable for thicker lesions owing to improved penetration depth. In their investigation, lesion thickness was defined by invasion depth, distinguishing thin tumors (≤1 mm) from thick tumors (>1 mm) [33]. Within this framework, the present study provides evidence that 20 MHz HFUS represents a pragmatic compromise, offering sufficient axial resolution for clinically meaningful depth assessment while maintaining adequate penetration across a broad spectrum of tumor depth. This balance is particularly relevant for routine clinical practice, where lesion depth can vary substantially and access to UHFUS systems may be limited. Nevertheless, it is important to note that further increases in ultrasound frequency, while improving spatial resolution, do not necessarily eliminate systematic discrepancies between ultrasound-based and histopathologic tumor depth measurements.

An additional contribution of the present study is the derivation of a preliminary depth-correction equation to estimate histopathologic tumor depth from 20 MHz HFUS measurements. The observed linear relationship (*R*^2^ = 0.99), alongside the low mean absolute error, indicates that HFUS-derived depth can approximate histopathologic depth with reasonable precision in this pilot cohort. From a clinical standpoint, this finding underscores the potential utility of calibration-based approaches to improve preoperative depth estimation and to reduce between-method discrepancies in routine HFUS assessments of BCCs.

Several limitations should be acknowledged. The relatively small sample size reflects the pilot nature of the study and limits statistical power, thereby limiting meaningful subgroup analyses according to histopathologic subtype, anatomical location, or lesion depth. Different BCC subtypes may influence ultrasound delineation due to variations in growth pattern and margin definition. However, the limited representation of individual subtypes in this cohort did not permit statistically robust subtype-stratified agreement analysis. Moreover, peritumoral features such as inflammatory infiltrate or stromal remodeling, which may affect ultrasound boundary definition and contribute to measurement variability, were not systematically evaluated in this pilot cohort. In addition, histopathologic depth measurement is itself subject to variability related to tissue orientation, sectioning plane, and processing-related dimensional changes (including fixation-induced shrinkage), which may influence agreement estimates. Furthermore, the use of a portable high-frequency ultrasound device in routine clinical settings may involve technical factors related to the gel interface, probe positioning, and focal depth adjustment, which can influence the visualization and overall image quality of very superficial skin structures. Finally, the proposed depth-correction equation was derived and evaluated within a single cohort, and therefore warrants validation in larger, independent populations prior to broader clinical application.

Despite these limitations, the present findings contribute to the growing body of evidence supporting HFUS for preoperative depth assessment in BCCs. When interpreted alongside existing studies spanning a wide ultrasound frequency spectrum, our results indicate that clinically acceptable depth estimation can be achieved using standard 20 MHz HFUS, supporting its use as a practical, accessible, and non-invasive modality in oncologic dermatology. At the same time, the persistent between-method differences reported across frequencies suggest that residual discrepancies are not fully mitigated by increased resolution alone. In this context, the agreement-based framework and preliminary depth-correction equation proposed here address an important unmet need by quantitatively calibrating HFUS measurements toward histopathologic depth, with the aim of improving individual-level accuracy and promoting more standardized clinical interpretation.

## 5. Conclusions

Twenty MHz HFUS demonstrated clinically acceptable agreement with histopathology for tumor depth assessment in BCCs. HFUS may serve as a useful, non-invasive tool for preoperative evaluation of BCCs in routine practice. The proposed depth-correction equation provides a preliminary method to calibrate HFUS-derived depth estimates toward histopathologic depth, although validation in larger, independent cohorts is required.

## Figures and Tables

**Table 1:** Clinical and histopathologic characteristics of BCCs included in the plot study and comparison of tumor depth measurements obtained by HFUS and histopathologic examination.

Lesion No.	Sex	Age (Years)	Anatomic Location	Histopathological Subtype	HFUS Depth (mm)	Histopathologic Depth (mm)
1	M	50	Inner canthus	Nodular BCC	2.6	2.0
2	M	58	Temporal region	Nodular BCC	18.0	20.0
3	F	77	Lumbar region	Nodular BCC	14.0	15.0
4	M	79	Ear	Adenoid BCC	7.0	6.1
5	M	68	Anterior thorax	Superficial BCC	3.5	3.0
6	F	62	Lumbar region	Infiltrative BCC	0.46	1.0
7	M	49	Nasal apex	Nodular BCC with adenoid cystic areas	4.6	4.1
8	M	73	Zygomatic region	Nodular BCC	8.96	10.0
9	F	76	Nasal ala	Infiltrative BCC	6.38	6.0
10	F	76	Upper eyelid	Nodular BCC with adenoid cystic areas	2.85	2.6
11	F	70	Scalp	Superficial BCC	3.3	3.0
12	M	84	Inner canthus	Nodular BCC	3.1	2.5
13	F	77	Posterior thorax	Nodular BCC	10.9	11.2
14	M	85	Zygomatic region	Nodular BCC	1.9	2.1
15	F	68	Infraorbital region	Superficial BCC	1.1	1.4
16	F	73	Nasal ala	Superficial BCC	1.5	1.2

## Data Availability

The original contributions presented in this study are included in the article. Further inquiries can be directed to the corresponding authors.

## Abbreviations

The following abbreviations are used in this manuscript:

BCC	Basal cell carcinoma
HFUS	High-frequency ultrasound
MAE	Mean absolute error
RMSE	Root mean square error
R2	Coefficient of determination
SD	Standard deviation
UHFUS	Ultra-high-frequency ultrasound
